# Self-Inflicted Male Bladder Foreign Body: Its Endoscopic Removal Using a Rigid Cystoscope and a Suprapubic Forceps

**DOI:** 10.1155/2013/729013

**Published:** 2013-02-27

**Authors:** Mohammad Kazem Moslemi, Mohamad Sorani

**Affiliations:** ^1^Department of Urology, Kamkar Hospital, School of Medicine, Qom University of Medical Sciences, Qom 3715694978, Iran; ^2^Tehran University of Medical Sciences, Tehran, Iran

## Abstract

*Introduction*. Herein we present an interesting technique for the removal of bladder foreign body (BFB) in which a combination of endoscopic and suprapubic cystostomy was used. *Case Presentation*. The patient was a case of illicit drug abuser who self-introduced an electrical wire into his bladder. After its failed cystoscopic removal, the foreign body was removed suprapubically without open bladder surgery. *Discussions*. Bladder foreign bodies are not uncommon. Based on the literature review, mainly open surgeries were used for their treatment. Using of our less invasive technique is a good way for escaping from open cystostomy. *Conclusion*. Endoscopic removal of the bladder foreign bodies is possible without any need for open bladder surgery.

## 1. Case Report

**Figure 1:**
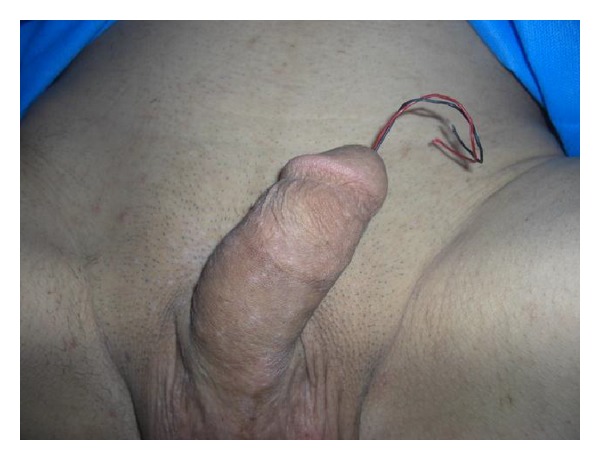
The wire ends are extruded from the external meatus.

**Figure 2:**
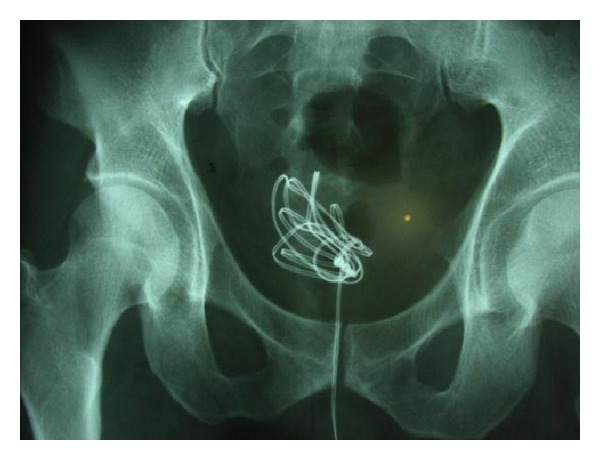
Pelvic X ray: the intravesical wire is evident.

**Figure 3:**
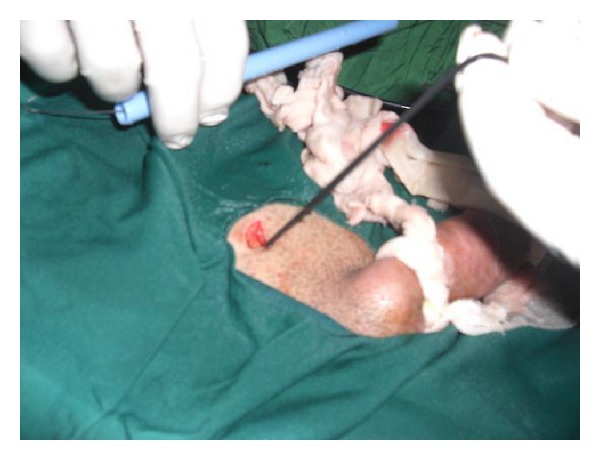
The ends of wire were remove from the suprapubic incision.

**Figure 4:**
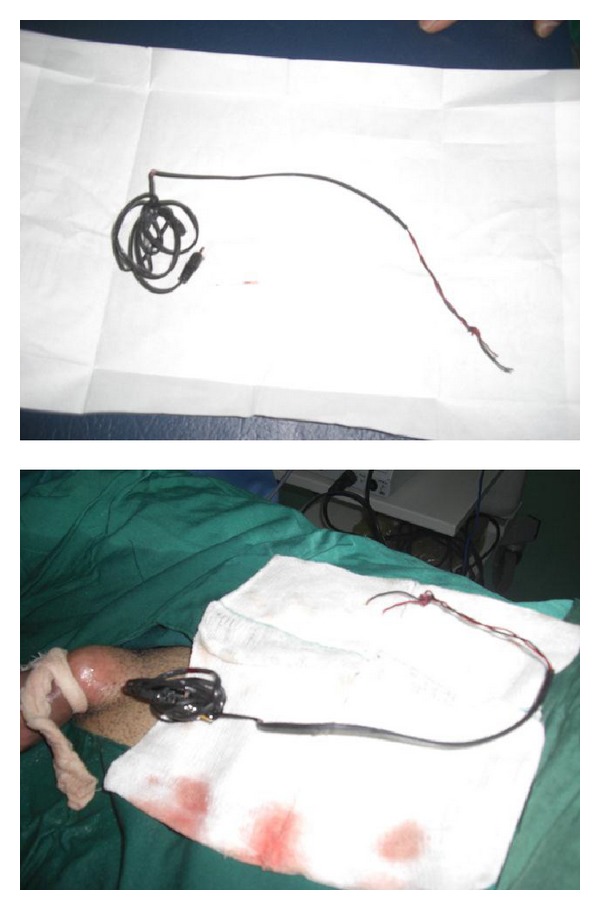
The removed electrical wire in its entire length.

The patient was a 47-year-old Iranian man who was admitted to the urology ward due to a self-inflicted foreign body, a cell phone charge wire. He was a victim of Iraq-Iran's war since 20 years ago. He was a case of treated posttraumatic stress disorder (PTSD). Also, he was an active consumer of some illicit drugs of unknown chemical formula. He self-introduced a cell phone wire that is used for the cell phones charges in its entire length for sexual pleasure. In physical examination, the end of wires was evident in the patient's meatus (Figure 1). Its removal manipulation was unsuccessful. An anteroposterior pelvic X-ray was performed, and the wire was seen curled up completely inside the bladder (Figure 2). The patient was scheduled for its removal. After induction of endotracheal general anaesthesia and under lithotomy position, rigid cystourethroscopy was performed. The foreign body removal with foreign body grasper was unsuccessful. Then a curved Boogie was introduced into the bladder transurethrally. Its tip was pointed to the suprapubic areas surface, and then a skin incision was made with a scalpel. The curved tip of Boogie was pushed to the outside of the bladder through skin incision. An Allis clamp grasped the Boogie tip forcefully, and finally the Allis clamp was pushed to the bladder. A 2° Fr rigid cystoscope (Storz, Tutlingen, Germany) with its obturator was introduced to the bladder transurethrally and it was filled with 300 mL of normal saline. The end of wire tips was grasped by the Allis clamp with the guidance of cystoscope and under direct vision, and finally the wire was extracted in its whole length suprapubically (Figures 3 and 4). The overlying fascia was closed with nylon suture. A Foley catheter was inserted transurethrally and was fixed for 24 hours. Then, the catheter was removed, and the patient was discharged home uneventfully with normal micturition ability. Close followup was performed by the treating psychiatrist.

## 2. Discussion

Bladder foreign bodies (BFBs) have an important role in the differential diagnosis of lower urinary tract symptoms (LUTSs) [1]. The etiology of bladder foreign bodies may include iatrogenic, urethral self-insertion, penetrating trauma, and migration from adjacent organs [1–5]. The most common reason for self introduction BFB is sexual gratification [1]. In some cases psychogenic problem may have an underlying influence such as in our case. The variety of objects as diverse as electric wire, glass rod, battery, pencil, toys, light bulbs, safety pins, and blue tracks have been reported [6–10]. Some objects are introduced by urologists such as catheters and endoscopic instruments [1]. Patients may present with acute or chronic symptoms due to the nature or the way of introduction of foreign bodies. The common symptoms include frequency, dysuria, hematuria, incontinency, external genitalia swelling, and acute urinary retention (AUR) [1]. Radio-opaque BFBs are easily detectable by kidney, bladder, and ureter (KUB) radiography. Abdominopelvic ultrasonography may be of help for the detection of nonradio-opaque materials [11, 12]. Cystourethroscopy is the most accurate method for the confirmation of the presence of the BFBs [1] and also is the most adequate treatment method [3]. Rafique M [1] studied a series of 16 patients and found that self-introduction for sexual pleasure was the cause in 25% of studied cases. Endoscopic removal of foreign bodies was done in 50% of cases, and the remaining was treated by open surgery. 

Most BFBs that are placed transurethrally are amenable to cystoscopic removal. We described a novel approach for removal BFBs in the cases of failed cystoscopic removal because of shape or size. It is a modification of Alagiri and Seidmon approach [13] for the placement of a suprapubic tube in the anterior bladder wall. We used a curved Boogie with a full bladder for the elevation of anterior bladder wall instead of a flexible cystoscope as a light source and a finder needle for the suprapubic cystostomy placement. There is one similar report in the literature that described a technique similar to our technique for removal of a BFB [10]. The difference is that we used a Boogie in which an Allis clamp grasped it, and entrance to the bladder is successful. But in the Delair et al. [10] report, he used the cut for the light technique, and the bladder was entered with the guidance of the finger. This mentioned way used by Delair et al. [10] is more invasive and the bladder surface may lose at the time of finger penetration. In addition, cystostomy catheter is needed. Our bladder incision is very small, and no cystostomy catheter is needed at all. 

## 3. Conclusion

The present report describes an interesting technique in which combination of a transurethral approach and mini-incisional suprapubic technique was used to avoid open surgery. Cystoscope, Allis clamp, and Boogie were used for removal of a curled up intravesical electrical wire. Using this technique is therefore appropriate for the removal of small- and medium-sized bladder stones.
